# Evaluation of the reporting quality of observational studies in master of public health dissertations in China

**DOI:** 10.1186/s12874-020-01116-6

**Published:** 2020-09-11

**Authors:** Shuangyang Dai, Xiaobin Zhou, Hong Xu, Beibei Li, Jingao Zhang

**Affiliations:** 1 Department of Epidemiology and Health Statistics, School of Public Health, Qingdao University, No. 38 Dengzhou Road, Qingdao, Shandong China; 2 Department of orthodontics, The affiliated hospital of Qingdao University, School of Stomatology, Qingdao University, Qingdao, 266021 China

**Keywords:** Dissertation, Evaluating, Master of public health, Observational studies, Reporting quality

## Abstract

**Backgrounds:**

Master of public health (MPH) plays an important role in Chinese medical education, and the dissertations is an important part of MPH education. In MPH dissertations, most are observational studies. Compared with randomized controlled trial (RCT), observational studies are more prone to information bias. So, the reporting of the observational studies should be transparent and standard. But, no research on evaluating the reporting quality of the MPH dissertation has been found.

**Methods:**

A systematic literature search was performed in the Wanfang database from January 1, 2014 to May 31, 2019. The Strengthening the Reporting of Observation Studies in Epidemiology (STROBE) statement was adopted to evaluate the reporting quality of the selected studies. Articles that met the following criteria were selected: (1) observational studies, including cross-sectional studies, case-control studies, and cohort studies; (2) original articles; (3) studies on humans, including both adults and children.

**Results:**

The Median of compliance to individual STROBE items was 74.79%. The mean (standard deviation) of STROBE score was 14.29 (1.84). Five items/sub-items were 100% reported (“reported” and “partly reported” were combined): background, objectives, study design, report numbers of individuals at each stage, and key result. Fifteen items/sub-items were reported by 75% or more. Reporting of methods and results was often omitted: missing data (6.67%), sensitivity analyses (3.63%), flow diagram (15.15%), and absolute risk (0%). Logistic regression analysis indicated that cohort studies (OR = 3.41, 95% CI = 1.27–9.16), funding support (OR = 4.37, 95% CI = 1.27–9.16) and more published papers during postgraduate period (OR = 3.46, 95% CI = 1.40–8.60) were related to high reporting quality.

**Conclusion:**

In short, the reporting quality of observational studies in MPH’s dissertations in China is suboptimal. However, it’s necessary to improve the reporting of method and results sections. We recommend that authors should be stricter to adhere STROBE statement when conducting observational studies.

## Background

Public health in the twenty-first century faces problems that are very different from those in previous centuries [1]. With the economic development in China, an increasing number of public health problems are appearing, which poses a serious challenge to public health practitioners [2, 3]. As of 2014, the total number of professional staff in Chinese public health institutions was only 87.5 million, which was well below the target of reaching 95 million in 2015. In addition, only 4.2% of public health professionals had a postgraduate degree [4]. The low quantity and insufficient quality of health professionals have hindered the development of public health services in China. Therefore, it is urgent to optimize training programmes to train more highly educated, application-oriented public health personnel. To fulfill this requirement, the Ministry of Education of China launched the full-time master of public health (MPH) postgraduate programme in 2009 [5]. Although the dissertation is an important part of MPH education, studies on the quality of these dissertations are still limited [6].

The randomized controlled trial (RCT) has been advocated as the gold standard for evaluating causal effects in medical studies. However, it is difficult to verify many studies by RCTs due to various ethical problems and side effects of intervention in practice [7–9]. Well-designed observational studies can not only provide abundant clues for investigating the causal relationship between exposure and diseases but also be more suitable for investigating the long-term and rare side effects of treatment modalities. Moreover, one study showed that approximately 90% of the papers published in medical journals are observational studies [10].

Compared with RCTs, observational studies cannot randomly assign study factors to the participants. They can rely only on comprehensive, objective descriptions or well-designed programmes to analyse, compare and summarize population phenomena and further explore the causal relationships between disease and exposure factors. Hence, the reporting of observational studies should be transparent and complete. Standardizing the reporting of observational studies can not only help editors and reviewers of medical journals to better understand the study designs but also provide important information for readers in related fields so that they can clearly understand the content and results of the research and improve their professional skills. Several incipient studies on reporting quality recognized deficiencies in medical studies, but all of these were limited by incomplete reporting quality evaluation standards [11–14].

In 2008, the Strengthening the Reporting of Observation Studies in Epidemiology (STROBE) statement was published to “improve transparency in reporting of observational studies” [15–18]. The STROBE statement contains a total of 22 items, including evaluations of research design, data collection, analytical techniques, and potential deviations. Since the STROBE statement was published, several studies have evaluated the reporting quality of clinical medicine articles and found that the reporting of observational studies needs to be improved [19–23].

At present, no research has been performed to evaluate the reporting quality of the dissertation for the Chinese MPH degree. Therefore, we used the STROBE statement to evaluate the reporting quality of observational studies in MPH dissertations in China, identify factors associated with high-quality reporting, and provide direction for writing the MPH dissertation.

## Methods

### Search strategy

We searched the relevant studies in the Wanfang database. The Wanfang database contains mainly Chinese dissertation and journal papers, including all the dissertations of higher education institutions or scientific study institutions that are approved for granting the MPH degree. The language was limited to Chinese, and the search strategy was (theme:(“cohort studies” OR “cohort analyses” OR “case-control studies” OR “case-control analyses” OR “cross-sectional studies” OR “prevalence studies” OR “current situation studies”) *profession:(Master of Public Health) * degree:(master)) * Date:2014–2019.

### Study selection

Articles that met the following criteria were selected: (1) observational studies, including cross-sectional studies, case-control studies, and cohort studies; (2) original articles; and (3) studies on humans, including both adults and children. The exclusion criteria were as follows: (1) review articles; (2) case reports; (3) quasi-randomized trials, randomized controlled trials and other interventional studies; and (4) articles for which the database provided only abstracts and not full texts.

The articles retrieved were preliminarily reviewed on the basis of titles and abstracts by two investigators independently. Any disagreement was resolved by consulting a senior author. After the initial screening, the full texts of the relevant research were searched, and the two investigators determined the final content of the literature review based on the inclusion and exclusion criteria.

### Data extraction

The extraction of data from the included articles was performed independently by the two investigators. The general information extracted included publication time, type of study, number of papers published during master studies, funding support, and number of statistical methods used. This information was extracted from each article. For dissertations, the publication time and funding support were indicated on the cover page. In China, all graduate students must list the titles of their own published paper at the end of the dissertation, from which we extracted the number of published papers. Published papers were not limited in terms of country, language, and database, but the papers published after graduation are not included in this information. The number of number of statistical methods used was collected by a “common statistical methods in medical studies” checklist (supplement.1).

### Quality assessment

On the basis of the detailed item descriptions of the STROBE statement, the reporting appraisal was performed by two investigators. Differences between the two investigators were resolved by discussion with the senior author until all differences were resolved. The STROBE statement contains 22 items: title and abstract (item 1), introduction (items 2 ~ 3), method (items 4 ~ 12), results (item 13 ~ 17), discussion (items 18 ~ 21), and other information (item 22). A score of 1 was assigned to items for which all the detailed information was reported, a score of 0.5 was assigned to items for which the detailed information was partly reported, and a score of 0 was assigned to items for which none of the information was reported. For items with sub-parts, fractional points were assigned depending on the number of sub-items met. Sub-item 6b is applicable only to match studies, and 14c is applicable only to cohort studies. Sub-items were removed from the denominator if they were not applicable [24]. Therefore, every study had an overall STROBE score rated from a maximum score of 22. To simplify the statistical analysis, we combined the “reported” and “partly reported” categories to calculate the “reporting rate” when describing the reporting rate of items.

### Data analysis

The continuous data subjected to normal distribution were presented as the mean and standard deviation (SD). Categorical variables were expressed as numbers and percentages. Comparisons of STROBE scores between dichotomous groups were conducted using the independent Student’s t-test. Comparisons of STROBE scores between multiple groups were conducted using one-way analysis of variance (ANOVA) with the LSD-t test. The included articles were further divided into high and low reporting quality groups according to the cut-off value (the 75th percentile of the STROBE score). Univariate logistic regression models were used to analyse the associations between high reporting quality and study type, publication time, papers published during master studies, funding support, and types of statistical methods used. Candidate variables for which *P* ≤ 0.05 in the univariate logistic regression analyses were included in the multivariate logistic regression model. The odds ratio (OR) and 95% confidence interval (CI) were calculated from the logistic regression analyses. Statistical analyses were performed using SPSS version 18.0. All reported probabilities (*P* values) were two-sided, and *P* ≤ 0.05 was considered significant.

## Results

### Search results

**Fig. 1:**
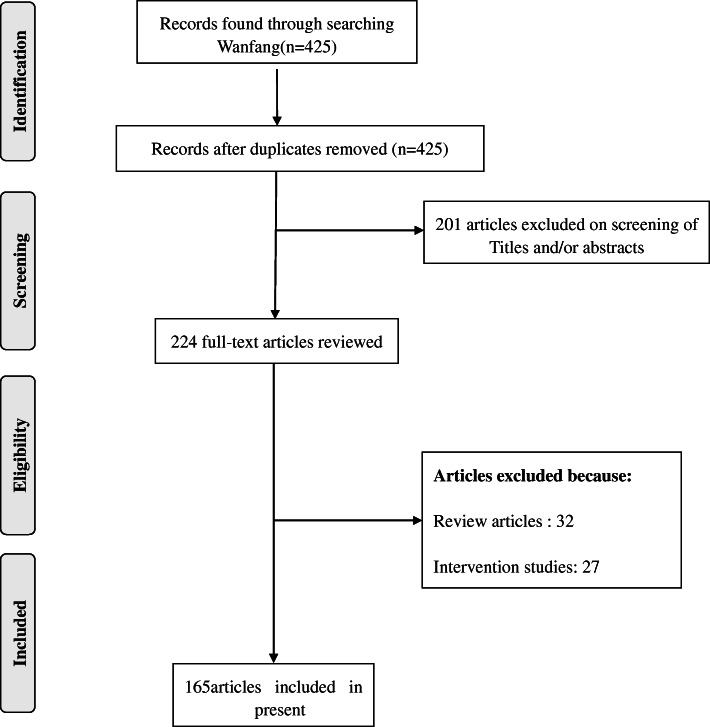
Flow diagram of the literature

After the search of the database, we confirmed 425 articles without duplication. After the titles and abstracts were screened, 201 articles were excluded. A total of 224 full articles were further reviewed, and 59 additional articles were excluded because 32 articles were review studies and 27 were intervention studies. Finally, 165 relevant articles that met the inclusion criteria were included. Of the 165 articles, 61 articles were cross-sectional studies, 66 articles were case-control studies, and 38 articles were cohort studies (Fig. 1).

### The compliance of the included studies with the STROBE statement

**Fig. 2:**
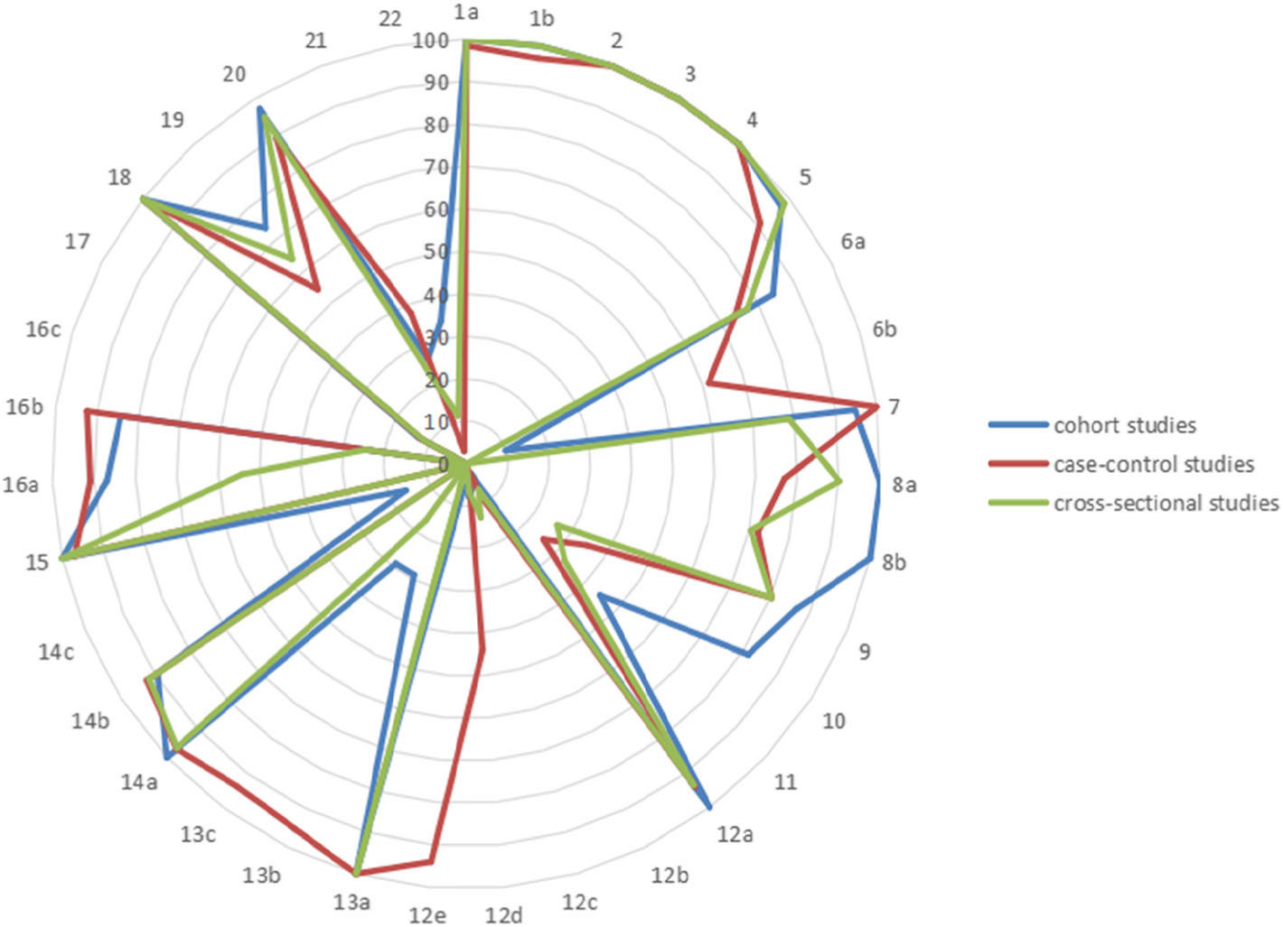
The compliance of three study types in each item

The adherence to the 22 items of the included studies is shown in Table 1. The median of compliance with individual STROBE items was 74.79% (range: 0–100%). The overall reporting quality was relatively good. Five items/sub-items (14.71%) were reported by 100% of the studies: background/rationale, objectives, study design, reported numbers of individuals at each stage of the study, and key results; 17 items/sub-items (50.00%) were reported by 75% or more of the studies. Specifically, the reporting on the title and abstract section and the introduction section was satisfactory: reporting rates for all four items exceeded 95%. Reporting on methods and results was often omitted: examine sub-groups and interactions (*n* = 11, 6.67%), missing data (*n* = 11, 6.67%), sensitivity analyses (*n* = 6, 3. 63%), reasons for non-participation (*n* = 14, 8.48%), flow diagram (*n* = 25, 15.15%), and absolute risk (*n* = 0, 0%). The reporting rates of the items were similar in the three study types, but the reporting of the cohort studies was slightly better (Fig. 2).

**Table 1:** Adherence to the STROBE Reporting Criteria

Item		Recommendation	Fully Reported (%)	Partly Reported (%)
**Title and abstract**	1	(a) Indicat the study’s design with a commonly used term in the title or the abstract	164 (99.39)	0 (0)
		(b) Provide in the abstract an informative and balanced summary of what was done and what was found	163 (98.79)	0 (0)
**Introduction**				
Background/rationale	2	Explain the scientific background and rationale for the investigation being reported	158 (95.75)	7 (4.25)
Objectives	3	State specific objectives, including any prespecified hypotheses	165 (100)	0 (0)
**Methods**				
Study design	4	Present key elements of study design early in the paper	165 (100)	0 (0)
Setting	5	Describe the setting, locations, and relevant dates, including periods of recruitment, exposure, follow-up, and data collection	129 (78.18)	28 (16.97)
Participants	6	(a) Cohort study—Give the eligibility criteria, and the sources and methods of selection of participants. Describe methods of follow-up Case-control study—Give the eligibility criteria, and the sources and methods of case ascertainment and control selection. Give the rationale for the choice of cases and controls Cross-sectional study—Give the eligibility criteria, and the sources and methods of selection of participants	89 (53.94)	31(23. 64)
		(b) Cohort study—For matched studies, give matching criteria and number of exposed and unexposed Case-control study—For matched studies, give matching criteria and the number of controls per case	41(73.21)	0 (0)
Variables	7	Clearly define all outcomes, exposures, predictors, potential confounders, and effect modifiers. Give diagnostic criteria, if applicable	52 (31.52)	98 (59.39)
Data sources	8	For each variable of interest, give sources of data and details of methods of assessment zhg (measurement). Describe comparability of assessment methods if there is more than one group	127 (76.97)	17 (10.30)
Bias	9	Describe any efforts to address potential sources of bias	135 (81.82)	0 (0)
Study size	10	Explain how the study size was arrived at	70 (42.42)	0 (0)
Quantitative variables	11	Explain how quantitative variables were handled in the analyses. If applicable, describe which groupings were chosen and why	21 (12.73)	33 (20.00)
Statistical methods	12	(a) Describe all statistical methods, including those used to control for confounding	141 (85.45)	16 (9.70)
		(b) Describe any methods used to examine subgroups and interactions	11(6.67)	0 (0)
		(c) Explain how missing data were addressed	11(6.67)	0 (0)
		(d) Cohort study—If applicable, explain how loss to follow-up was addressed Case-control study—If applicable, explain how matching of cases and controls was addressed Cross-sectional study—If applicable, describe analytical methods taking account of sampling strategy	36 (21.82)	0 (0)
		(e) Describe any sensitivity analyses	6 (3. 63)	0 (0)
**Results**				
Participants	13	(a) Report numbers of individuals at each stage of study—eg numbers potentially eligible, examined for eligibility, confirmed eligible, included in the study, completing follow-up, and analysed	49 (29.70)	116 (70.30)
		(b) Give reasons for non-participation at each stage	14 (8.48)	0 (0)
		(c) Consider use of a flow diagram	25(15.15)	0 (0)
Descriptive data	14	(a) Give characteristics of study participants (eg demographic, clinical, social) and information on exposures and potential confounders	79 (47.88)	82 (49.70)
		(b) Indicate number of participants with missing data for each variable of interest	14 (8.48)	0 (0)
		(c) Cohort study—Summarise follow-up time (eg, average and total amount)	6 (15.79)	0 (0)
Outcome data	15	Cohort study—Report numbers of outcome events or summary measures over time Case-control study—Report numbers in each exposure category, or summary measures of exposure Cross-sectional study—Report numbers of outcome events or summary measures	163 (98.79)	0 (0)
Main results	16	(a) Give unadjusted estimates and, if applicable, confounder-adjusted estimates and their precision (eg, 95% confidence interval). Make clear which confounders were adjusted for and why they were included	58 (35.16)	68 (41.21)
		(b) Report category boundaries when continuous variables were categorized	26 (15.76)	0 (0)
		(c) If relevant, consider translating estimates of relative risk into absolute risk for a meaningful time period	0 (0)	0 (0)
Other analyses	17	Report other analyses done—eg analyses of subgroups and interactions, and sensitivity analyses	20 (12.12)	0 (0)
**Discussion**				
Key results	18	Summarise key results with reference to study objectives	165 (100)	0 (0)
Limitations	19	Discuss limitations of the study, taking into account sources of potential bias or imprecision. Discuss both direction and magnitude of any potential bias	87 (52.73)	16 (9. 69)
Interpretation	20	Give a cautious overall interpretation of results considering objectives, limitations, multiplicity of analyses, results from similar studies, and other relevant evidence	137 (83.03)	18 (10.91)
Generalisability	21	Discuss the generalisability (external validity) of the study results	45 (27.27)	2 (1.21)
**Other information**				
Funding	22	Give the source of funding and the role of the funders for the present study and, if applicable, for the original study on which the present article is based	20 (12.12)	0 (0)

Note:The compliance of 6b refer to the compliance of match studies (*n* = 56, 73.21 = 41/58*100%). The compliance of 14c refer to the compliance of cohort studies (*n* = 38, 15.79% = 6/38*100%)

“Reported” and “partly reported” was combined as adherence

### STROBE score

The mean of the STROBE score was 14.29 (range: 10.03–18.93) with a standard deviation of 1.84. We found that the STROBE score of the cohort studies was significantly higher than that of the other two types of studies. Dissertations with funding support were more likely to receive high STROBE scores. The STROBE scores of dissertations that listed more papers published during the postgraduate period and more statistical methods was higher than that of others. The mean of the STROBE score of dissertations published in 2018 was higher than that of those published in other years. The characteristics of the 165 included articles are shown in Table 2.

**Table 2:** The main characteristics of included studies

Characteristic	N (%)	Mean (SD)	*t/F*	*P*
Type of study				
Cohort studies	38 (23.03)	15.58 ± 1.70^*^		
Case-control studies	66 (40.00)	13.77 ± 1.56	14.53	< 0.001
Cross-sectional studies	61 (36.97)	14.05 ± 1.86		
Funding support				
No	143 (87.27)	14.15 ± 1.79	−2.41	0.017
Yes	21 (12.73)	15.17 ± 2.01		
Number of published papers during postgraduate period				
< 2	60 (36.36)	13.86 ± 1. 67	−2.37	0.019
≥ 2	105 (63.64)	14.56 ± 1.90		
Number of the statistical methods				
< 3	73 (44.24)	13.80 ± 1.77	−3.12	0.012
≥ 3	92 (55.76)	14.68 ± 1.81		
Year of published				
2014	33 (20)	14.29 ± 1.83		
2015	48 (29.10)	13.80 ± 1.89		
2016	47 (28.48)	14.31 ± 1.61	2.57	0.040
2017	31 (18.79)	14.71 ± 1.89		
2018	6 (3.64)	15.94 ± 1.84^*^		

Note:*P < 0.05compared with other groups

### Univariate and multivariate logistic regression analyses

According to the cut-off value of the STROBE score (15.40), the included articles were divided into low (*n* = 124) and high reporting quality groups (*n* = 41). The univariate logistic regression analyses showed that the following factors were related to superior reporting quality: cohort study (OR = 4.08, 95% CI = 1.67–10.00), funding support (OR = 2.71, 95% CI = 1.05–7.02), more papers published during the postgraduate period (OR = 2.52, 95% CI = 1.11–5.73), and number of statistical methods used (OR = 3.33, 95% CI = 1.46–7.56).

The multivariate logistic regression analysis demonstrated that cohort studies (OR = 3.41, 95% CI = 1.27–9.16), funding support (OR = 4.37, 95% CI = 3.52–7.48), and more papers published during the postgraduate period (OR = 3.46, 95% CI = 1.40–8.60) were related to superior reporting quality (Table 3).

**Table 3:** Univariate and Multivariate Logistic Regression Analyses of Predictive Factors Associated With Superior Reporting Quality

	Univariate		Multivariate	
Variables	OR (95%CI)	*P*	Adjusted OR (95%CI)	*P*
Type				
Cross-sectional studies	1			
Case-control studies	0.73 (0.29–1.84)	0.502	0.66 (0.25–1.77)	0.410
Cohort studies	4.08 (1. 67–10.00)	0.002	3.41 (1.27–9.16)	0.015
Funding support				
No	1			
Yes	2.71 (1.05–7.02)	0.040	4.37 (3.52–7.48)	< 0.001
Number of published papers during postgraduate period				
< 2	1			
≥ 2	2.52 (1.11–5.73)	0.028	3.46 (1.40–8.60)	0.007
Number of the statistical methods				
< 3	1			
≥ 3	3.33 (1.46–7.56)	0.004	1.893 (0.74–4.74)	0.183
Year	1.32 (0.96–1.82)	0.088		

Note: The included articles were divided into superior and inferior reporting quality groups according to the cut-off value (the 75 percentile of the STROBE score, 15.40)

## Discussion

### Summary of findings

Our study evaluated 165 MPH dissertations. Although the overall reporting quality was relatively good, some essential aspects of methods and results were seldom reported, which makes it difficult for readers to assess the validity and reliability of an observational study [16]. Moreover, dissertations of superior reporting quality usually contained the following predictive factors: cohort study, funding support and more papers published during the postgraduate period.

Reporting on the title and abstract section and the introduction section was satisfactory. The reason may be that each MPH candidate needs to undergo strict opening and midpoint screening stages in the early stage of the dissertation writing. The deficiency of the reporting of MPH dissertations occurred mainly in methods and results. In particular, there was a need for dissertations to improve their reporting of variable definitions, statistical methods, and flow diagrams.

In actual studies, the outcome, exposure, predictors, potential confounders, and effect modifiers of the study should be clearly defined, but less than half of the dissertations fully reported these contents. Inadequate reporting of statistical methods may indicate that the research results are not fully exploited, resulting in a waste of valuable information and varying degrees of bias. However, only a few articles described any methods used to examine sub-groups and interactions, explained how missing data were addressed, and described any sensitivity analysis. Only 25 dissertations (15.15%) used flow diagrams, while others did not take advantage of the simple and direct features of the flow diagram. In addition, all of the articles summarized the key results with reference to the study objectives, but only approximately one-quarter of the articles discussed the generalizability of the study results.

The results of the multivariate logistic regression analysis indicated that funding support was associated with high reporting quality. To receive funding, projects require rigorous research designs and need to be screened and approved. Therefore, masters candidates are strictly required and trained to learn more knowledge to ensure that their thesis quality will be higher. Moreover, a positive association between more papers published during the postgraduate period and high reporting quality was observed. Masters candidates who published more papers during the postgraduate period have stronger academic ability, are more familiar with writing articles, and know what should be reported in detail. In addition, the results of the univariate logistic regression analyses showed that a higher number of statistical methods was associated with high reporting quality. Masters candidates who use more statistical methods have a deeper understanding of methodology, are more proficient in using statistical methods, and tend to be more complete in reporting their methods in their dissertations.

### Compared with other studies

A few articles have evaluated the reporting quality of observational studies in other medical disciplines. Several studies have found that the reporting quality of articles that used the STROBE statement for standardization was better than that of others [19, 20, 25, 26]. Jacqueline Ramke et al. used the STROBE statement to evaluate reporting in blindness prevalence surveys and found that the mean of the STROBE score of studies published in journals requiring the STROBE statement was higher than that for others [19]. Swords C’s study indicated that the STROBE statement had increased the reporting quality of observational otology and audiology studies [20]. Hence, we strongly recommend that masters candidates should be familiar with the STROBE statement.

On the other hand, many studies have found defects in reporting the methods and results of observational studies [27–29]. Adams AD et al. discovered poor reporting in obstetrics observational studies for study size, missing data, and absolute studies [27]. Karaçam Z evaluated the reporting quality of observational studies in Turkish nursing journals and found that methods sections were mostly omitted [29]. Our research yielded similar results.

### Educational implications

Our study has highlighted the important deficiencies in the reporting of observational studies in MPH dissertations. Based on these findings, we believe that if universities adopt the STROBE criteria to guide MPH candidates, it will help improve the reporting quality of MPH dissertations. In the course of master training, it is necessary to strengthen students’ understanding and flexible application of statistical methods, and graduate tutors should pay more attention to masters students who published fewer papers during the postgraduate period.

### Strengths and limitations of this study

As a systematic review of MPH dissertations, our study has some advantages. First, it is a comprehensive assessment and used logistic regression analyses to identify factors associated with high-quality reporting. For the evaluation of dissertations, we included not only adherence to STROBE items but also the STROBE score. Second, some of the evaluation items, such as 6b and 14c, are not applicable to all dissertations, and some items are not adequately reported. To minimize biases against systematic review, we identified items as fully reported, partly reported, not reported, and not applicable and assigned the scores accordingly. Thus, different articles have a more consistent score criterion. Third, since the STROBE statement was published in 2007, no studies have used this guideline to evaluate the reporting quality of master dissertations. Therefore, our work is innovative and will provide a reference for subsequent similar research. Fourth, the study includes the independent assessment of all articles by two authors. All details of our search are transparent and clearly stated, and it can therefore easily be reproduced.

There are also some limitations of this study. First, the scoring of items remains a subjective task and easily leads to subjective bias. However, the two investigators independently used the STROBE statement to evaluate the included studies, and differences were resolved by discussion. In this way, we minimized subjective bias. Second, given that our research was restricted to MPH dissertations published by Chinese masters candidates in the past 5 years, the results reflect only the integrity and standardization of the reporting of Chinese MPH dissertations to a certain extent. Finally, since there is no literature to be found on using the STROBE statement to evaluate the reporting quality of medical masters dissertation, it is impossible to compare the reporting quality of these dissertations with that of dissertations in other professions.

## Conclusion

In summary, the reporting quality of observational studies in MPH dissertations is sub-optimal. There is a need to improve the reporting of methods and results sections, especially statistical methods reporting. The STROBE statement was intended to help researchers to improve transparency in reporting observational studies. Therefore, we think it is highly plausible that using the STROBE statement will improve the quality of reporting. We recommend that masters candidates who conduct observational studies use the STROBE statement and recommend that research supervisors use this statement to guide MPH candidates.

## Supplementary information

**Additional file 1.**

## Data Availability

None.

## Abbreviations

MPH: Master of public health
RCT: Randomized controlled trial
STROBE: Strengthening the Reporting of Observation Studies in Epidemiology statement
OR: Odds ratios
CI: Confidence intervals

## References

[1] Begg MD (2014). MPH education for the 21st century: design of Columbia University's new public health curriculum. Am J Public Health.

[2] Blair SN (2009). Physical inactivity: the biggest public health problem of the 21st century. Br J Sports Med.

[3] Li W, Chen B, Ding X (2012). Environment and reproductive health in China: challenges and opportunities. Environ Health Perspect.

[4] NHaFPCotPsRo, C (2015). 2015 china health statistics yearbook.

[5] Wang N, Wang Y, Jia J, et al. The investigation on current status of cultivation of full-time MPH students in China. Chin J Med Educ Res. 2015;3:232–6.

[6] Katikireddi SV, Reilly J (2017). Characteristics of good supervision: a multi-perspective qualitative exploration of the masters in public health dissertation. J Public Health (Oxf).

[7] Chen Y (2010). Assessment of the quality of reporting in abstracts of randomized controlled trials published in five leading Chinese medical journals. PLoS One.

[8] Cho HJ (2013). Assessments of the quality of randomized controlled trials published in international journal of urology from 1994 to 2011. Int J Urol.

[9] McIntyre A (2014). The evolution of stroke rehabilitation randomized controlled trials. Int J Stroke.

[10] Stang A, Kantelhardt E (2013). Too many statistical errors for meaningful interpretation. Breast Cancer Res Treat.

[11] Wolf FM (2004). Methodological quality, evidence, and research in medical education (RIME). Acad Med.

[12] Price EG (2005). A systematic review of the methodological rigor of studies evaluating cultural competence training of health professionals. Acad Med.

[13] Cook DA, Beckman TJ, Bordage G (2007). Quality of reporting of experimental studies in medical education: a systematic review. Med Educ.

[14] Howley L (2008). Quality of standardised patient research reports in the medical education literature: review and recommendations. Med Educ.

[15] Vandenbroucke JP (2007). The making of STROBE. Epidemiology.

[16] von Elm E (2014). The strengthening the reporting of observational studies in epidemiology (STROBE) statement: guidelines for reporting observational studies. Int J Surg.

[17] von Elm E (2007). The strengthening the reporting of observational studies in epidemiology (STROBE) statement: guidelines for reporting observational studies. Lancet.

[18] Cuschieri S (2019). The STROBE guidelines. Saudi J Anaesth.

[19] Ramke J (2017). Using the STROBE statement to assess reporting in blindness prevalence surveys in low and middle income countries. PLoS One.

[20] Swords C (2019). An assessment of the change in compliance of observational otology and audiology studies with the STROBE statement guidelines: a systematic review. Otol Neurotol.

[21] Sorensen AA (2013). Using the strengthening the reporting of observational studies in epidemiology (STROBE) statement to assess reporting of observational trials in hand surgery. J Hand Surg Am.

[22] Irani M (2018). Weaknesses in the reporting of cross-sectional studies in accordance with the STROBE report (the case of congenital anomaly among infants in Iran): a review article. Iran J Public Health.

[23] Serrano M (2014). Adherence to reporting guidelines in observational studies concerning exposure to persistent organic pollutants and effects on semen parameters. Hum Reprod.

[24] Agha RA (2016). Reporting quality of observational studies in plastic surgery needs improvement: a systematic review. Ann Plast Surg.

[25] Pouwels KB (2016). Quality of reporting of confounding remained suboptimal after the STROBE guideline. J Clin Epidemiol.

[26] Rao A (2016). Quality of reporting and study design of CKD cohort studies assessing mortality in the elderly before and after STROBE: a systematic review. PLoS One.

[27] Adams AD (2018). Use of the STROBE checklist to evaluate the reporting quality of observational research in obstetrics. Obstet Gynecol.

[28] Wang YT (2017). Quality analysis of observational studies on pelvic organ prolapse in China. Zhonghua Fu Chan Ke Za Zhi.

[29] Karacam Z, Sen E, Yildirim B (2015). Evaluation of observational research reports published in Turkish nursing journals. Int Nurs Rev.

