# Supplementary material for: Evaluation of the reporting quality of observational studies in master of public health dissertations in China
Source: BMC Med Res Methodol. 2020 Sep 11;20:230. doi: 10.1186/s12874-020-01116-6 (PMC7488525; doi:10.1186/s12874-020-01116-6)
Supplement: Supplementary file 1 — Additional file 1. [file 12874_2020_1116_MOESM1_ESM.docx]

**Common statistical methods in medical studies**

| Descriptive statistics |  |  |
| --- | --- | --- |
|  | Means (standard deviation) |  |
|  | Median (interpercentile range) |  |
|  | Proportion |  |
|  | Rate (e.g. incidence rate, survival rate) |  |
|  | Ratio (e.g. odds ratios, relative risk) |  |
| Ancillary analyses |  |  |
|  | Variable transforms |  |
|  | Variable constructs |  |
|  | Standardizing |  |
|  | Matching |  |
|  | Propensity score methods |  |
|  | Sensitivity analysis |  |
|  | Stratification or sub-group analyses |  |
| Student *t*-test |  |  |
|  | One sample *t*-test |  |
|  | Paired/matched *t*-test |  |
|  | Two independent samples *t*-test |  |
|  | *Z* test |  |
| Analysis of variance(ANOVA) |  |  |
|  | Completely random design ANOVA |  |
|  | Randomized block design ANOVA |  |
|  | Factorial design ANOVA |  |
|  | Cross-over ANOVA |  |
|  | Analysis of covariance |  |
|  | Multivariate Analysis Of Variance |  |
| Multiple comparisons |  |  |
|  | Students-Newman-Keuls method |  |
|  | Bonferroni method |  |
|  | Dunnett method |  |
|  | Duncan's method |  |
|  | LSD method |  |
|  | Tukey method |  |
|  | Sidak method |  |
|  | Scheffe method |  |
|  | FDR (false discovery rate) |  |
| Repeated measurement data |  |  |
|  | Repeated measurement data ANOVA |  |
|  | GEE (Generalized estimating equation) |  |
|  | MMRM (Mixed-effect models for repeated measures) |  |
|  | GLMM (generalized linear mixed models) |  |
| Non-parametric test |  |  |
|  | Sign test |  |
|  | Wilcoxon signed-rank test |  |
|  | Mann-Whitney test |  |
|  | Kruskal-Wallis *H* test |  |
|  | Friedman test |  |
|  | Kolmogorov-Smirnov test |  |
|  | Median test |  |
| Contingency tables |  |  |
|  | Chi-square test |  |
|  | McNemar’s test |  |
|  | Fisher’s exact test |  |
| Correlation analysis |  |  |
|  | Pearson correlation coefficient |  |
|  | Spearman correlation coefficient |  |
|  | Kendall’s correlation coefficient |  |
|  | Trend test |  |
|  | Partial correlation coefficient |  |
|  | Multiple correlation coefficient |  |
| Multiple regression |  |  |
|  | Linear regression |  |
|  | Curve estimate |  |
|  | Path analysis |  |
|  | Logistic regression |  |
|  | Poisson regression |  |
|  | Negative binominal |  |
|  | Spline regression |  |
|  | Other regression model |  |
| Survival analysis |  |  |
|  | Kaplan-Meier estimate |  |
|  | Life-table method estimate |  |
|  | Log-rank test |  |
|  | Breslow test |  |
|  | Tarone-Ware test |  |
|  | Cox proportional hazards model |  |
|  | Other survival model |  |
| Consistency measurement |  |  |
| Principal component analysis |  |  |
| Factor analysis |  |  |
| Discriminant analysis |  |  |
| Cluster analysis |  |  |
| Log-linear models |  |  |
| Structural equation modeling (SEM) |  |  |
| Multilevel modeling |  |  |
| Multi dimensional scaling analysis |  |  |
| Bayesian analyses |  |  |
| Other statistical methods |  |  |
